# How to win in FIFA World Cup Qatar 2022? A study on the configurations of technical and tactical indicators based on fuzzy-set qualitative comparative analysis

**DOI:** 10.3389/fpsyg.2023.1307346

**Published:** 2024-01-26

**Authors:** Weihua Yan, Shiyue Li, Di Wang, Bo Yuan, Haocheng Zeng, Dingmeng Ren

**Affiliations:** ^1^China Football College, Beijing Sport University, Beijing, China; ^2^Zhongshan No. 1 Middle School, Zhongshan, China

**Keywords:** football, FIFA World Cup, Qatar 2022, performance analysis, qualitative comparative analysis (QCA), technical and tactical indicators, configuration

## Abstract

The FIFA World Cup, which represents the highest level in football, is regarded as a showcase to unfold the development trends of modern football, thus arousing great interest among researchers. However, most of the previous research designs studied the simple linear correlation between technical indicators and game outcomes, which may overlook the complex causalities in football performance. The aim of current study was to introduce a new method to examine winning patterns emerging from Qatar 2022 through a configurational lens. To this end, fuzzy-set qualitative comparative analysis (fsQCA) was conducted using 98 samples (*n* = 98) out of 49 Qatar 2022 matches discriminating winning and losing teams in regular time (group stage) and in 30 min of extra time (knockout stage). Then, we selected seven variables as our causal conditions, namely, shots on target, possession, defensive line breaks, crosses, receptions in the final third, forced turnovers, and direct pressures. Necessity analysis and sufficiency analysis of configurations were conducted according to fsQCA requirements. The fsQCA operation showed that no individual causal condition is necessary to winning a game and four configurations were derived from the QCA results and these combinations of conditions fall into three typologies of play style: a possession play style, direct play style, and all-round play style. The results confirmed the fact that football is a complex system and suggested that a winning outcome is often produced by combinations of multiple factors. The findings of the current study contribute to the literature by introducing the configurations of various technical and tactical indicators that could raise the possibility of winning and can be used by practitioners working within the fields of player development, coaching, and match preparation.

## 1 Introduction

Teams participating in the quadrennial FIFA World Cup are top national squads from all over the world and represent the highest level in football; therefore, the FIFA World Cup is a showcase to unfold the development trends of modern football (Yi et al., 2019). To win every single game is the ultimate goal of teams that have the great honor of being involved in the World Cup. To this end, besides the coaches, players, supporting staff, etc., that work very hard as a team, scholars also make efforts to explore the winning patterns and new trends hidden behind the FIFA World Cup games of the latest version from the perspective of technical and tactical performance (Wallace and Norton, 2014; Rumpf et al., 2017; Vergonis et al., 2019).

Most of the previous research designs have concentrated on logistic regression and studied the simple linear correlations between one or various technical indicators and the game outcomes (Tenga et al., 2010; Collet, 2013; Liu et al., 2015). However, the combination relationships between technical and tactical factors have not received sufficient attention. A correlational approach is useful for identifying simple “best practices” (Delery and Doty, 1996), like more ball possession being better than less ball possession; nevertheless, it also tends to favor elegance over realism (Howell et al., 2022) since examining linear correlations between indicators and the game outcomes can be ambiguous. For instance, much research has supported the idea that possession is conducive to positive game results (Lago-Peñas et al., 2011; Göral, 2015) while others have not (Stanhope, 2001; Hughes and Franks, 2005; Collet, 2013). As another example, different studies from different perspectives may also yield divergent results regarding the role played by passing in match-play (Scoulding et al., 2004; Redwood-Brown, 2008).

Field sports like football involve multifaceted, complex, and non-linear dynamical systems (Mackenzie and Cushion, 2013; Wallace and Norton, 2014). One single indicator cannot solely decide the result of match-play, and it can only take effect with the presence of other supporting indicators. That is, there is often a complex concurrent causal relationship between technical and tactical performance and match results; therefore, an alternative approach is warranted (Mackenzie and Cushion, 2013). Given the complexity of football games and the mixed findings regarding the research results, the qualitative comparative analysis (QCA) approach enables us to find equifinal yet asymmetric combinations of various technical and tactical indicators, thus revealing the complex configurations leading to winning outcomes.

QCA was firstly used in humanities and social sciences, the studies of which are always full of complexity, and a single condition or cause cannot explain the complex mechanisms behind phenomena. Realizing the importance of set theory to the fields of humanities and social sciences, the sociologist Ragin pioneered the QCA method in 1987 (Ragin, 1987). Based on set theory and Boolean algebra, QCA attempts to go beyond the traditional case study techniques and systematically investigates the combination and interaction relationships between attributes under which the outcome occurs using quantitative data in order to deepen the understanding of the multiple concurrent causal relationships between causal conditions (i.e., explanatory variables) and results (Kumar et al., 2022). Multiplicity refers to the number of paths towards the same goal, and concurrency means that each path is composed of different combinations of attributes. At the same time, the data of different types across cases can be analyzed and compared by calibration techniques, so this method for bringing together basic concepts from both qualitative and quantitative techniques of analysis differs substantially from traditional methods of quantitative analysis that are often variance-based (Pappas and Woodside, 2021).

QCA mainly includes three branches: crisp-set qualitative comparative analysis (csQCA), fuzzy set qualitative comparative analysis (fsQCA), and multi-value qualitative comparative analysis (mvQCA). Through fsQCA, we can overcome the shortcomings of traditional methods based on the “independent variable—dependent variable” binary relationship and study the interdependence of causal conditions and the multiple concurrency causation composed of different combinations (Rihoux and Ragin, 2008). Therefore, we employ the fsQCA technique and adopt a configurational perspective to examine the effect of different indicators on game outcomes in latest FIFA World Cup Qatar 2022 (Qatar 2022).

Specifically, the aim of this study is to identify the overarching configurations of various technical and tactical indicators that could raise the possibility of winning in the FIFA World Cup Qatar 2022 (Qatar 2022). In particular, we explore the questions of:

- Which technical and tactical factor(s) is (are) necessary to win a match?
- What configurations of technical and tactical indicators can lead to a winning outcome?

## 2 Methods

### 2.1 Applicability of QCA to our study design

FsQCA is used because it offers a series of analytical advantages relevant to this study, namely, conjunction, equifinality, and symmetry.

#### 2.1.1 Conjunction

An important reason why football has become the most popular sport and attracts hundreds of millions of fans around the world is that this sport is an organic social system where interactions between team members result in varied intra-team coordination patterns of play (Passos et al., 2011). The significance of technical and tactical superiority to the victory of the game is needless to say, so scholars have paid full attention to this aspect of research. However, existing studies often explore the “net effect” of a given variable on the outcome by controlling irrelevant variables, that is, a simple linear correlation between a single independent variable and the dependent variable, such as the impact of possession control on the outcome of a game. A complex system means no one factor can determine the result alone. Instead, the performance of a football squad depends largely on the consistency or internal fit among several elements that are organically combined. In this regard, configurational theory helps understanding how multiple conditions may combine in complex ways (Misangyi et al., 2017; Furnari et al., 2021).

#### 2.1.2 Equifinality

Configurational methodology highlights that more than one combination of conditions can lead to the same outcome and searches for multiple ways to explain the same phenomenon (Howell et al., 2022). The complex system of football has produced a rich variety of technical and tactical combinations, which represent different playing styles, such as: the Argentine style of delicate technology and flexible transmission, the Spanish style that advocates possession control and rhythm, and the French style that focuses on counterattacking and pragmatism. Different styles have different technical and tactical demands, but all these different styles of play could equally lead to the positive outcome of a game. In a word, there are multiple combinations of indicators for the positive outcome of a football match. But some styles are more in line with the trends of modern football, while others are gradually becoming outdated.

#### 2.1.3 Asymmetry

For the same outcome, a given causal condition may be related in one configuration but unrelated in another configuration. In the case of a football match, to explore the importance of a certain indicator is in vain, because several key factors combined could lead to desired outcomes and a particular variable may not have explanatory power given the complexity of this sport. Traditional correlational study is challenged in understanding how different attributes might combine in complex ways (Howell et al., 2022), while FsQCA is well-suited to accommodate such complexities because it allows for causal asymmetry between conditions and outcomes (Witt et al., 2022). This may explain to some extent why researchers cannot find an agreement regarding the role of some indicators on game results since they study these factors separately.

### 2.2 Sample selection

The data of the current study were derived from the FIFA official website database (FIFA Training Centre) where all the post-match summaries of Qatar 2022 can be found (FIFA, 2022a). These summaries were conducted by a panel of seven experts led by Arsène Wenger who observed all the tournament’s matches and reflected on matches straight after the final whistle (FIFA, 2022b). It is known that Qatar 2022 comprised 64 games with 64 pairs of opponents, thus our initial sample contained 128 cases. However, not all these cases can be included in our study. The inclusion criteria of our study dictated that sample selection should clarify the purpose of the research, that is, to explore the winning law of Qatar 2022 and meet the requirements of the fsQCA method. Based on this, the current study selected 49 games discriminating winning and losing teams in regular time (group stage) and in 30 min of extra time (knockout stage). Fifteen matches in which the winner was decided by penalty shoot-out were excluded from our study given that penalty shoot-outs are more related to the footballers’ shooting techniques, psychological quality, and penalty kick strategies (McGarry and Franks, 2000; Vandebroek et al., 2018) so have nothing to do with the purpose of this research. Therefore, 49 matches means 49 pairs of opponents, so in the end there were 98 samples included in our research.

Past research suggests that this medium-N sized cases/samples meet the criteria used for QCA studies (Ragin and Fiss, 2008; Rihoux and Ragin, 2008), as it is small enough to allow for an in-depth familiarity with cases, and large enough to allow a systematic investigation of the relationships between causal conditions that emerge from the data. What is more, selection bias was avoided since all the tournament games were taken into consideration except for tied games that obviously did not fit.

### 2.3 Variable selections

#### 2.3.1 Outcome condition

Scoring more goals than the opposing team is the essence of a football competition and winning is the ultimate objective for the players and coaching staff on and off the field. Therefore, we directly used the match result as our outcome condition. The outcome can be coded as 1 or 0 in fsQCA, which mean fully in winning set or fully out of winning set respectively. However, the dichotomous way of coding results neglects the difference in huge victories and narrow victories (for instance, winning by 4:0 is different from winning by 1:0). Therefore, the difference of scores between opponents is adopted as an outcome variable, and with calibration techniques, the continuity of data and the difficulty of winning can be included in our study design. Specifically, in a match, the fact that the number of goals scored by the home team minus that scored by the away team is a positive number means the home team wins. If the calculation result is zero, it means that the two sides tie. For example, the largest score difference in Qatar 2022 was seven, with Spain beating Costa Rica by 7:0, which means that in this game Spain is coded as 7 in terms of result value while Costa Rica is coded as −7.

#### 2.3.2 Causal conditions

The causal conditions in this study were derived from the Key Statistics of the FIFA World Cup post-match summaries. Sometimes one causal condition includes more than one indicator that work together, in our case, one causal condition equals one indicator. Performance indicators are defined as the selection and combination of variables that define some aspects of performance and help achieve athletic success (Hughes and Bartlett, 2002). With the development of science and technology, especially with the application of artificial intelligence, big data, and other new technologies, the technical and tactical data of the World Cup are becoming more and more abundant. However, a great deal of variables are neither possible nor desirable when using QCA (Misangyi et al., 2017) because the combinations of conditions can be too complicated and it is too hard to discover dominating patterns that lead to the expected result. Therefore, the following principles are formulated to select causal conditions.

First, football is practice-oriented but also needs theoretical guidance, so an inductive approach is suitable to select causal conditions (Howell et al., 2022). That is to say, we can derive conditions from a mix of extant theory and previous literature (Lago-Peñas et al., 2010; Yi et al., 2019). Especially, we should focus on a variety of attributes that past literature has deemed important or controversial. Second, both in-possession and out-of-possession variables should be taken into account in accordance with the real game situation. Therefore, the causal conditions should include variables related to goals scoring, variables related to offense, and variables related to defense that are in line with previous studies (Lago-Peñas et al., 2011; Yi et al., 2019) (Table 1). On the other hand, the selected conditions should be directly related to the results of the competition, while physical and situational variables are not primary considerations. Third, we must pay attention to indicators that are newly included into match summaries by FIFA. Fourth, the number of technical and tactical indicators should fit the number of conditions that can be accommodated by QCA. Considering these inclusion criteria, we selected seven variables as our causal conditions.

**Table 1:** Definitions of causal conditions.

Categories	Causal conditions	Definitions
Variables related to goal scoring	Shots on target	A player has an attempt at goal and the ball ends inside the goal frame without any external influences on its route.
Variables related to offense	Possession control	Possession control shows the percentage of time each team is in possession of the ball, as well as a new third state showing when the ball is in contest.
	Crosses	A distribution action performed by a player with the intention of creating a goal scoring opportunity. The player can play the ball on the ground or aerially from any crossing zone with the intention of finding a team-mate inside the recognized target area.
	Defensive line breaks	An opposition line is broken when the attacking team play the ball beyond the deepest player in that line. Once the defensive line is broken, attacking teams must show efficiency in attempts at goal.
	Receptions in the final third	Final third entries show where on the pitch a team develops its attacking play as they approach an opponent’s goal. It also exposes potential weaknesses in an opponent’s defensive unit and structure.
Variables related to defense	Direct pressures	A player has directly and aggressively closed down space between themselves and the opposition player with the ball and can compete for possession.
	Forced turnovers	Forced turnovers show when possession is lost due to pressure from an opponent. The more pressure teams and players apply to an opponent, the more likely they are to force a turnover of possession.

From https://www.fifatrainingcentre.com/.

##### 2.3.2.1 Shots on target

The ultimate objective of a football match is to win by scoring as many goals as possible; so, the primary factor that determines game result is teams’ ability to finish. There are three indicators in relation to scoring goals in the FIFA Post Match Summary Reports: goals, attempts at goal, and shots on target. Goals are the outcome condition of our study. Shots on target is adopted as one of our causal conditions instead of attempts at goal in consideration of the following reasons: for one thing, most of the previous studies found that winning teams had more attempts on target (Lago-Peñas et al., 2011; Dufour et al., 2017). For another, some studies have also demonstrated that total shots are of great importance to winning the match and shots on target per shot are not (Lago-Peñas et al., 2010; Lepschy et al., 2020). But a dispute exists because in practice, some players shoot simply trying to keep their state in order not to forget the feeling of scoring or have a good post-match report in terms of statistics. Therefore, shots on target may be a direct and objective indicator.

##### 2.3.2.2 Possession control

Possession control, namely, ball possession or simply possession, is a highly valued indicator for football practitioners and researchers influenced by Barcelona’s style of play. It has been suggested to be linked with success (Bloomfield et al., 2005) while others hold that this variable is a poor predictor in terms of match outcomes (Stanhope, 2001; Hughes and Franks, 2005; Collet, 2013). What is more, possession is believed to have a significant positive correlation with the amount of passes by a team (Tenga et al., 2010; Collet, 2013). “In contest” is a newly introduced part of “possession” in this edition of the Word Cup. In our study, this percentage was not taken into account since it means it is not clear which team possesses the ball.

##### 2.3.2.3 Crosses

Crosses are very frequent in a football match. Crosses could be the action that directly leads to a goal, so the importance of crosses to generate goal-scoring opportunities has been highlighted by previous studies (Pulling et al., 2018). Moreover, there is much evidence proving the effectiveness of crosses in determining the winning teams (Lago-Peñas et al., 2010, 2011). In light of the reasons mentioned above, crosses were included in our study.

##### 2.3.2.4 Defensive line breaks

Line breaks are new FIFA indicator that include completed line breaks and defensive line breaks. Putting the out-of-possession opponent under pressure does not necessarily mean goal-scoring because sometimes pressure is applied far away from the attacking third. Defensive line breaks are of greater importance (Smith and Lyons, 2017) considering they occur closer to the opponent’s goal and thus were included in our study.

##### 2.3.2.5 Receptions in the final third

The field of play can be divided horizontally into three parts: the back third, middle third, and final third (Smith and Lyons, 2017). The final third is the area nearest to opponent’s goal and the most dangerous area for defending squad and receptions in the final third could create direct scoring opportunities through finishing or indirect opportunities when an attacking player is fouled by opponents.

##### 2.3.2.6 Direct pressures

Defensive pressures are significantly associated with goal-scoring attempts and attacking outcomes (Pulling et al., 2018). Defensive pressures, especially direct pressures, can also prevent opponents from building up attacks and creating good scoring opportunities. For instance, empirical evidence revealed that crossing ball speed could be significantly reduced when defensive pressure was increased (Orth et al., 2014), so defenders and the goalkeeper can be better prepared for a clearance or block the ball. On the other hand, less defensive pressure on the receiver of a pass correlated with a higher pass completion rate of opponents (Herold et al., 2022) and the offensive players can have the time and space to better execute various skills (Duarte et al., 2012). Moreover, defensive errors can cause catastrophic situations for the team (Lepschy et al., 2020).

##### 2.3.2.7 Forced turnovers

Forced turnovers are a new technical and tactical indicator introduced by FIFA so few previous studies have concentrated on this indicator (Vella et al., 2022). This indicator is of great interest because, as a defensive variable, it also reflects the offensive and defensive conversion of the team. Therefore, we included this indicator as one causal condition.

### 2.4 Calibration

After selecting the outcome and causal conditions, the next step of the fsQCA technique is to calibrate the set membership by assigning values between 0 (indicating the absence of a given condition) and 1 (indicating the presence of the condition), depending on degree of membership. In theory, almost all the indicators in football fields are continuous numbers like “possession” changing from 0 to 100%, so we used fuzzy-set coding in our study. We employed the direct method to calibrate, that is, the 75th and 25th percentiles as fully-in or fully-out membership in the sets, respectively, and the 50th percentile as the crossover point (Fiss, 2011). Table 2 provides a detailed explanation of our calibration method.

**Table 2:** Calibration of set membership.

Conditions and outcome	Calibration anchor points		
	Full membership = 0.75	Crossover = 0.5	Non-membership = 0.25
Outcome	1.75	0	−1.75
Target	6	4	2
Possession	0.539	0.4505	0.352
DLB	12.75	8	7
Crosses	22	18	13
RFT	112.75	95.5	77.25
DP	54	48.5	39
FT	79	71.5	61.25

DLB, defensive line breaks; RFT, receptions in the final third; DP, direct pressures; FT, forced turnovers.

## 3 QCA findings

### 3.1 Necessity analysis

The necessary conditions should be identified prior to determining the configurations for an outcome (Schneider and Wagemann, 2012). In the current study, we can see whether a causal condition is necessary to winning the game by conducting the necessity analysis. When a condition can produce the outcome, that condition is a necessary condition for the outcome (Ragin and Fiss, 2008). That is to say, the goal of necessity analysis is to find out whether there is a/several condition(s) that is (are) necessary for the winning outcome to occur; contrarily, the result cannot be produced without the condition. It can also be absence of the/these condition(s) (i.e., the proportion of the outcome in which the probability of winning is high due to the absence of this condition).

Consistencies of 1.0 represent being always necessary (Ragin, 2008). Therefore, Ragin and Fiss (2008) suggested that the condition is necessary for the outcome when its consistency value is greater than 0.9. By using the software fsQCA 3.0 Necessary Analysis function, it was found that none of the above 14 single conditions is an inevitable or necessary condition for the outcome of winning (as shown in Table 3).

**Table 3:** Result of necessity analysis.

Causal conditions (~represents its non-set)	Outcome			
	Result		~Result	
	Consistency	Coverage	Consistency	Coverage
Target	0.652	0.710	0.383	0.417
~Target	0.464	0.429	0.733	0.678
Possession	0.522	0.524	0.561	0.563
~Possession	0.564	0.562	0.526	0.524
DLB	0.609	0.607	0.459	0.458
~DLB	0.456	0.458	0.606	0.608
Crosses	0.501	0.504	0.560	0.564
~Crosses	0.567	0.563	0.508	0.504
RFT	0.611	0.593	0.504	0.489
~RFT	0.473	0.488	0.580	0.599
DP	0.543	0.541	0.530	0.528
~DP	0.526	0.528	0.539	0.541
FT	0.575	0.601	0.457	0.478
~FT	0.501	0.480	0.618	0.593

### 3.2 Sufficiency analysis

Sufficiency analysis of the condition configurations is conducted from a causal perspective in order to determine whether there exist combinations of conditions (i.e., the intersection of causal conditions) that are sufficient for the desired outcome. From the perspective of set theory, it is discussed whether the set represented by a configuration and composed of multiple causal conditions is a subset of the outcome set. Sufficiency analysis is the essence of QCA methodology. Through the necessity analysis of each condition and the sufficient analysis of condition configurations, the current study can demonstrate the impact of individual factors and combinations of factors on the outcome.

The criterion for setting a threshold is that the row consistency value should be greater than 0.75 according to Schneider and Wagemann (2012). Moreover, considering the size of our samples, we set other parameters (see Table 4).

**Table 4:** Threshold settings in the fsQCA 3.0 software.

Frequency	Row consistency	PRI consistency
1	0.8	0.8

The results of QCA consist of three solutions, namely, a parsimonious solution, intermediate solution, and complex solution (Ragin, 2008). Our report is based on the intermediate solution and supplemented by the parsimonious solution (Fiss, 2011). Four combinations were derived from our QCA results, as shown by Table 5. The table displays the different combinations of conditions that can lead to winning outcomes. The circles in each column show whether a condition is present (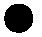) or absent () in the combination. QCA also differentiates between core conditions (denoted by larger circles), those that are present in both the parsimonious and intermediate solutions and are therefore central to the combination, and peripheral conditions (denoted by smaller circles), those occurring only in the intermediate solution.

**Table 5:** Configurations of technical and tactical indicators.

Causal conditions	Configurations			
	1	2	3	4
Target	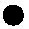	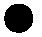	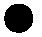	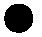
Possession	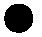	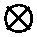		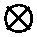
DLB	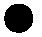	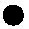	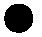	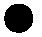
Crosses	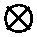	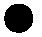	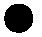	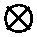
RFT	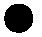	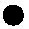	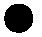	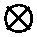
DP			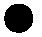	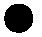
FT	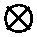	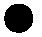	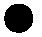	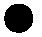
Raw coverage	0.109	0.114	0.116	0.060
Unique coverage	0.072	0.036	0.033	0.035
Consistency	0.897	0.925	0.926	0.899
Solution coverage	0.265			
Solution consistency	0.927			

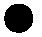
, core condition when present; 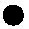, peripheral condition when present; 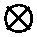, core condition when absent; 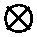, peripheral condition when absent; empty cells indicate indifference.

#### 3.2.1 Configuration 1

This QCA combination and case suggest that when lacking crosses and forced turnovers but having possession, defensive line breaks, and receptions in the final third, a team is likely to win in this edition of the World Cup, and shots on target plays a complementary role because it is a peripheral condition. FsQCA results show that the Consistency, Raw Coverage, and Unique Coverage of this configuration are 0.897, 0.109, and 0.072, respectively, which means with this configuration there is an 89.7% possibility to win the game, this combination can account for 10.9% of winning cases, and 7.2% of cases can only be explained by this configuration.

#### 3.2.2 Configuration 2

Shots on target, crosses, and forced turnovers combined play a central role in winning a match and defensive line breaks and receptions in the final third are peripheral conditions. Possession is absent as a core condition, meaning that it is not useful for positive game results in this combination. Direct pressures are of little significance. FsQCA results show that the Consistency, Raw Coverage, and Unique Coverage of this configuration are 0.925, 0.114, and 0.036, respectively, which means with this configuration there is a 92.5% possibility to win, this combination can account for 11.4% of winning cases, and 3.6% of cases can only be explained by this configuration.

#### 3.2.3 Configuration 3

Shots on target, crosses, direct pressures, defensive line breaks, receptions in the final third, and forced turnovers are central to winning the game. Possession is a matter of no account in this case. FsQCA results show that the Consistency, Raw Coverage, and Unique Coverage of this configuration are 0.926, 0.116, and 0.033, respectively, which means with this configuration there is a 92.6% possibility to win, this combination can account for 11.6% of winning cases, and 3.3% of cases can only be explained by this configuration.

#### 3.2.4 Configuration 4

Shots on target, defensive line breaks, forced turnovers, and direct pressures are the core conditions. On the other hand, possessions, crosses, and receptions in the final third are absent, meaning that they are not useful in terms of winning a game in this configuration. FsQCA results show that the Consistency, Raw Coverage, and Unique Coverage of this configuration are 0.899, 0.060, and 0.035, respectively, which means with this configuration there is an 89.9% possibility to win, this combination can account for 9% of winning cases, and 3.5% of cases can only be explained by this configuration.

## 4 Discussion

The results are discussed in the following order. First, we explore the reason why none of the causal conditions are necessary conditions to winning a game. Second, we discuss the results of the sufficiency analysis. We label the four configurations derived from QCA into three categories according to existing studies (Hughes and Franks, 2005; Kempe et al., 2014). In this step, we also compare the configurations with the existing theory and literature and return to cases to interpret the results of the QCA; thus, we take full advantage of QCA and find a third way beyond qualitative and quantitative methods.

### 4.1 Discussion on necessity analysis

None of the above 14 single conditions was necessary for the outcome of winning, with shots on target having the greatest consistency value of 0.652 in terms of winning the game and non-target having the greatest value of 0.733 in terms of losing the game. The results confirm the fact that football is a complex system and suggest that the winning outcome of a football match is often produced by combinations of multiple factors and that a team cannot win a match if it only excels in a specific indicator. Nevertheless, attempting to shoot is always important in a football game because it is strictly linked to a possible goal (Lago-Peñas et al., 2011; Fernandez-Navarro et al., 2016; Dufour et al., 2017). Methodically, the results show that traditional linear regression analysis that tries to find causality between a certain independent variable and the game result could be improved since it cannot reflect football as it is.

### 4.2 Discussion on sufficiency analysis

The most important aim of this study is to detect combinations of conditions that are of greatest value relative to winning outcomes. Differences in technical and tactical performance among different solutions could provide different ways of winning. Our study also provides three dominating winning match-play styles in Qatar 2022 derived from four configurations.

The first configuration could be labeled as a possession play style. Teams employing a possession-play tactical style have relatively more ball possession than their rivals, with longer ball possession time and more passes (Tenga et al., 2010). In this configuration, forced turnovers and crosses are absent; one possible reason to explain this phenomenon is that when a team possesses the ball for much longer than the opponents, it does not lose the ball too often, so the frequency of forced turnovers is not so high and that teams in this configuration have a strong control power over the situation so they create opportunities using technical superiority such as good passing techniques. They play forward, create more progressive passes, and score using the middle area of the field, which can be evidenced by the presence of defensive line breaks and receptions in the final third. The greater the chance a team has of overtaking the last defenders and entering the attacking third of the field, the more goal scoring opportunities can consequently be created. This finding is also evidenced by Smith and Lyons (2017) who demonstrated that passing the ball behind opponent defenders or to a player level with the last defender from a central area in front of the big box is a very good way of scoring in FIFA World Cups.

When tracing typical cases through QCA, we can find that winning teams using this pattern were Spain (against Costa Rica), Portugal (against Ghana), England (against Wales), Argentina (against Australia), etc. These teams are well-known for their sophisticated skills and comprehensive strength. Crosses are absent, because the teams are good at penetration and use the middle area more especially the open space between the middle area and the wings to create opportunities against their opponents.

The second scenario could be named as a direct play style, as opposed to the first one given that the condition “possession” is absent. In this pattern, winning teams do not have an advantage in terms of possession at all. However, these teams may have effective counterattacking from the wings (because crosses are a core condition and receptions in the final third are a peripheral condition) and shooting skills (shots on target is a core condition) when they retain the ball. Therefore, this direct play style is characterized by trying to move the ball into the attacking third as directly as possible as a means of counterattacking (Fernandez-Navarro et al., 2016).

Typical winners with this style were Iran (against Wales), Croatia (against Canada), and France (against Denmark). For example, France did not have an advantage in possession when facing Denmark, but registered more crosses (23 against 18), more shots on target (6 against 2), and more goals (2 against 1) which may be explained by the fact that Mbappé and Dembélé were wing forwards and Giroud was in the middle. Teams with this playing style should have more intelligence and speed to counterattack.

The third configuration could be labeled as an all-round play style since in this configuration six indicators are the core conditions except for possession which does not make a difference relative to the desired game result. Hence, the all-round play style means the winning teams have comprehensive competitive advantages over their opponents statistically. By comparison with the second and the fourth configurations, we can see the third configuration is the enhanced version since it comprises all the core and peripheral conditions of the second and the fourth configurations. Therefore, this pattern may be suitable for the team that has the absolute dominant position compared to its rivals. Typical winners using this pattern were Croatia (Croatia vs. Canada), Brazil (Brazil vs. Korea) and the USA (USA vs. Iran) according to QCA.

**Figure 1:**
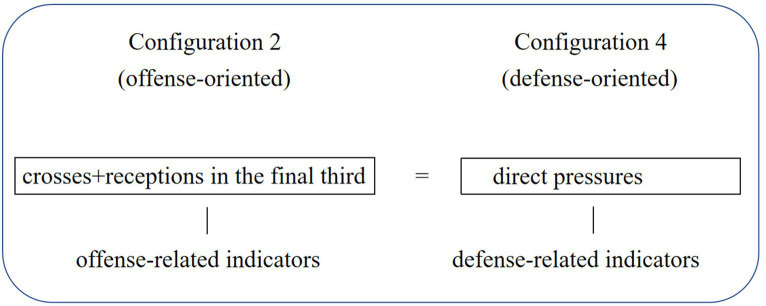
Replacement relationship between indicators.

The fourth configuration also falls into the category of a direct play style since “possession” is again absent and defensive line breaks are present. However, crosses and receptions in the final third are also absent relative to the second configuration while the defense is stronger considering that the two defense-related variables are present at the same time while only one variable related to defense is present in the second configuration. These disparities between the two direct play styles suggest that with shots on target, defensive line breaks, and forced turnovers remaining the same core conditions, the team can remedy its relatively weak wing attack and beat the opponent by reinforcing its defensive line breaks and more importantly, its defense (Figure 1). Moreover, the fact that two offense-related core conditions in this configurations are shots on target and defensive line breaks, with the other two being defensive indicators, suggests that once the ball goes beyond the opponent’s defensive line, offensive attempts have a higher possibility of being converted into a goal.

A representative case of this play style is Morocco beating Belgium by 2:0. By reviewing the game, we can find that Morocco had less possession (33%) and less crosses (9 against 19) but were very aggressive and solid in defense, based on which they built up their effective counterattacks and thus created two scoring chances. Morocco made penetrative passes in order to reach into the opposition penalty box (Ruiz-Ruiz et al., 2013) and had a better target/goal ratio since Morocco scored two goals out of four shots on target while Belgium worked in vain with no goal scored from four shots on target. Actually, Morocco registered good statistics in terms of all the defensive indicators no matter whether these indicators were included in our causal conditions or not: 78 forced turnovers against 38; 53 direct pressures against 36; and 360 defensive pressures applied against 194. This evidence also hints at the importance of defense in World Cup games. Teams can win against stronger opponents but the physical demands are greater when teams face stronger opposition (Ryan et al., 2017).

Numerous previous studies concentrated on the role played by possession and yielded contradictory conclusions. The answer to this question in our study is that possession is not that important in modern football because it is not a necessary condition of winning the match and it only appears once (in configuration 1) as a core condition across the four configurations. Without possession, a team also has the possibility to win the match if it excels in defense and is effective at counterattacking, as shown in configuration 4. On the contrary, configuration 1 demonstrates the condition in which teams with a possession play style can win, namely, to have “effective” possession, as Hughes and Franks (2005) showed that there were differences between successful and unsuccessful teams in converting possession into shots on goal, with the successful teams having better ratios. Instead, conducting too many unproductive, horizontal, or backward passes under ball control situation were predictive of worse team outcomes (Collet, 2013). To play a possession style, a team needs good technique.

All in all, configurations 2 and 4 are similar in terms of low possessions and great effectiveness, but the winning teams falling into configuration 2 have diversified ways to attack and score while the winners characterized by configuration 4 are better at defense and adopt a more typical counterattacking style. This disparity between the two categories may be due to the differences in the relative strength of the teams faced with their opponents. Compared to teams belonging to the fourth configuration, squads from the second configuration lose possession not due to weak strength; instead, they know how the tactical approach adopted in a specific match can be defined (Lago-Peñas et al., 2017) and how to create space when losing the ball and use limited ball possessions to attack.

Defense should always be highlighted in modern football since all the defense-related indicators are core conditions in configurations 3 and 4. Only in configuration 1 are all the indicators relating to defense absent; this phenomenon may be explained by the fact that winning teams falling into this configuration are good at possession so their data relative to defense are not outstanding compared to other teams.

Finally, it should be noted that shots on target is a very important indicator. Although it is not a necessary condition to winning the game, it appears in every configuration as a core or peripheral condition (Table 5).

Our study has several limitations, which may give orientations to future research. First, we strove to find winning patterns and technical and tactical features across winning teams through QCA but we did not consider conditions for rivals, and the importance of including the opposition in analysis should be highlighted (Tenga et al., 2010). Second, the outcome of a match is also linked to contextual elements such as weather, match locations, etc. However, in our study the match performance indicators were analyzed without taking into account situational elements. Third, we did not discuss further which one of the four configurations is more suitable for football games to date.

## 5 Conclusions and practical applications

### 5.1 Conclusion

The purpose of current study is to detect combinations of conditions that are of greatest importance relative to a winning outcome. All in all, QCA show that no one single condition is a necessary condition to win a game, which hints that the organic combination of indicators could lead to a winning outcome of football match. Our study provides four winning configurations that could fall into three dominating winning match-play styles in Qatar 2022, namely, possession play style, direct play style, and all-round play style. These combinations of indicators constitute a profile of ideal performance that led to positive game results in this edition of the FIFA World Cup. A team can take multiple paths to success and select one of these four patterns based on its own qualities and characteristics of its opponents. Moreover, according to evidence observed from this study, good defense, more attempts on goal, and effectiveness are suitable for tournaments like the FIFA World Cup.

We demonstrated the theoretical importance and broader significance of adopting a configurational perspective for both practitioners and scholars of football. A primary contribution of this paper is an emergent configurational methodology in football performance analysis instead of the linear analysis that we found in previous studies.

### 5.2 Practical applications

All sports evolve with time because of various reasons (Norton and Olds, 2001). The findings may indicate to some extent the future trends of modern football development and provide insights into the establishment of performance profiles that can serve as a reference for youth development and elite football.

On the one hand, the training of young players should be focused on improving ball kicking skills and finishing capacities in order to prepare for their grow-up stages. On the other hand, the results can support coaches from elite football teams in guiding training sessions and match preparations. Coaches have to focus on improving the technical and tactical build up into the attacking third or penalty area, encourage more goal attempts within the penalty box especially improving the ratio of converting attempts into goals, enhance the effectiveness of the team, and get the squad physically prepared for modern football games.

## Data availability statement

Publicly available datasets were analyzed in this study. This data can be found here: https://www.fifatrainingcentre.com/.

## Author contributions

WY: Writing – original draft. SL: Methodology, Software, Writing – review & editing. DW: Methodology, Writing – review & editing. BY: Formal analysis, Writing – review & editing. HZ: Formal analysis, Writing – review & editing. DR: Supervision, Writing – original draft.
